# Supplementary material for: VLX600, an anticancer iron chelator, exerts antimicrobial effects on Mycobacterium abscessus infections
Source: Microbiol Spectr. 2025 Jun 20;13(8):e00719-25. doi: 10.1128/spectrum.00719-25 (PMC12323642; doi:10.1128/spectrum.00719-25)
Supplement: Supplemental material — Tables S1 and S2; Fig. S1 to S6. [file spectrum.00719-25-s0001.pdf]

Subspecies (n)	ID number	Morphotype	Resistance-related genes				Susceptibility		
			rrl		***erm(41)***	***rrs***	**macrolides**		**aminoglycosides**
			**2058**	**2059**	**28**	**1408**	**intrinsic**	**inducible**	
abscessus (9)	5	Rough	A	A	T	A	susceptible	resistant	susceptible
	8	Smooth	A	A	T	A	susceptible	resistant	susceptible
	11	Rough	A	A	T	A	susceptible	resistant	susceptible
	16	Rough	A	A	T	A	susceptible	resistant	susceptible
	19	Rough	A	A	T	A	susceptible	resistant	susceptible
	23	Smooth	A	A	T	A	susceptible	resistant	susceptible
	28	Smooth	A	A	T	A	susceptible	resistant	susceptible
	37	Smooth	A	A	T	A	susceptible	resistant	susceptible
	40	Rough	A	A	T	A	susceptible	resistant	susceptible
massiliense (9)	26	Rough	A	A	-	A	susceptible	-	susceptible
	A56	Rough	A	A	-	A	susceptible	-	susceptible
	B115	Smooth	A	A	-	A	susceptible	-	susceptible
	B152	Rough	A	A	-	A	susceptible	-	susceptible
	C11	Rough	A	A	-	A	susceptible	-	susceptible
	E22	Rough	A	A	-	A	susceptible	-	susceptible
	E50	Rough	A	A	-	A	susceptible	-	susceptible
	G53	Rough	A	A	-	A	susceptible	-	susceptible
	H05	Rough	A	A	-	A	susceptible	-	susceptible

Supplemental Table 1. The list of clinical isolates used in the study and their profiles.

	PBS	VLX600 (5 mg/kg)	VLX600 (10 mg/kg)	VLX600 (5 mg/kg) + Amikacin (50 mg/kg)	Amikacin (50 mg/kg)
**Strain 1**	**15333**	**7167**	**2833**	**1867**	**1350**
**Strain 2**	**5833**	**6167**	**2666**	**1833**	**5000**
**Strain 3**	**14667**	**4333**	**5000**	**3167**	**2500**
**Strain 4**	**10833**	**4000**	**4167**	**8167**	**7167**
**Strain 5**	**20333**	**9333**	**2333**	**7500**	**1333**

Supplemental Table 2. Raw data of CFU counts in mouse lungs. All lung lobes except for the postcaval lobe, were homogenated in PBS. The homogenates were serially diluted in PBS and plated in triplicate on 7H10 agar plates. Colonies were enumerated after incubation, and the values from triplicate were averaged.

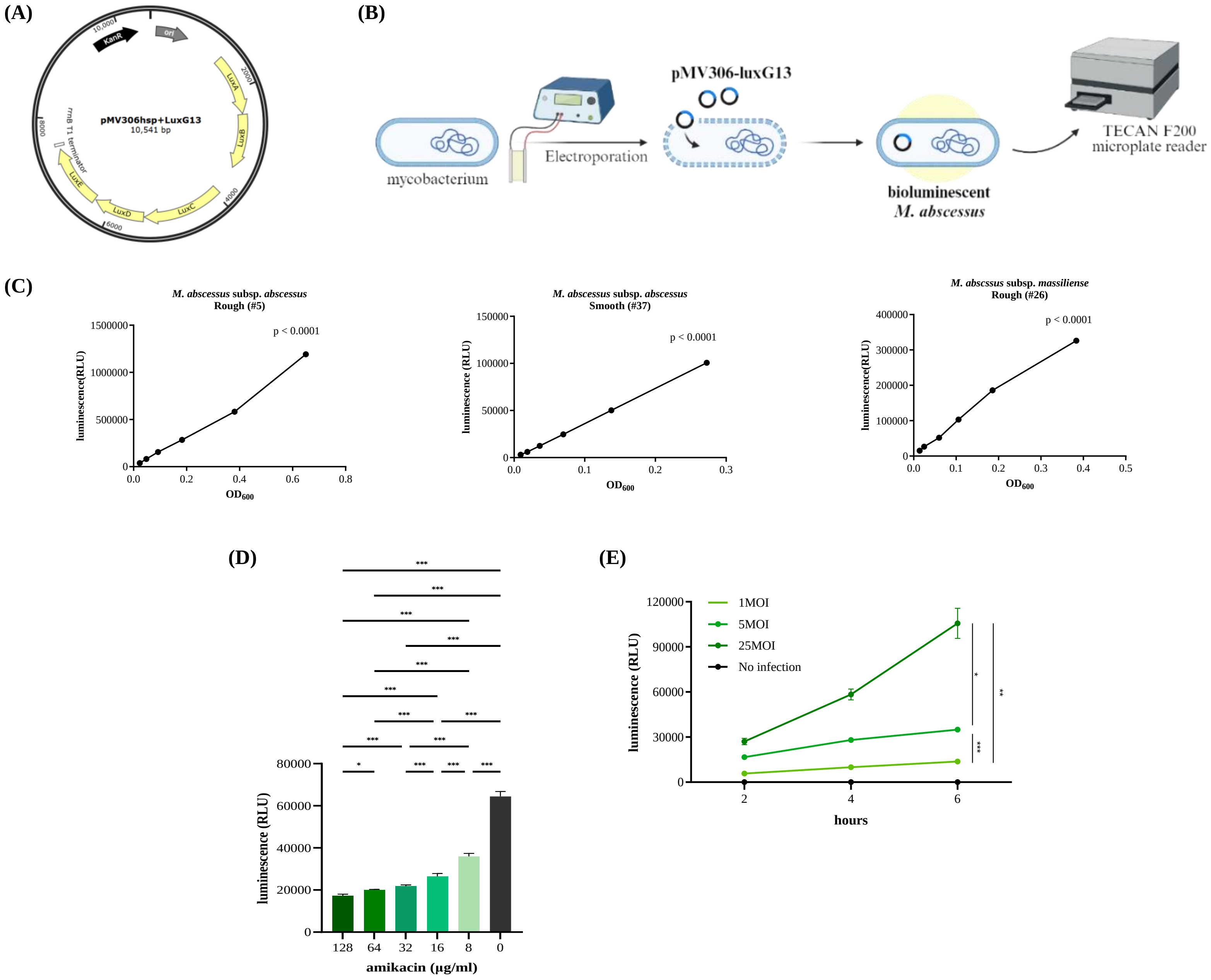

Supplemental Figure 1. Construction of bioluminescent *M. abscessus* strains and validation as a viability reporter.

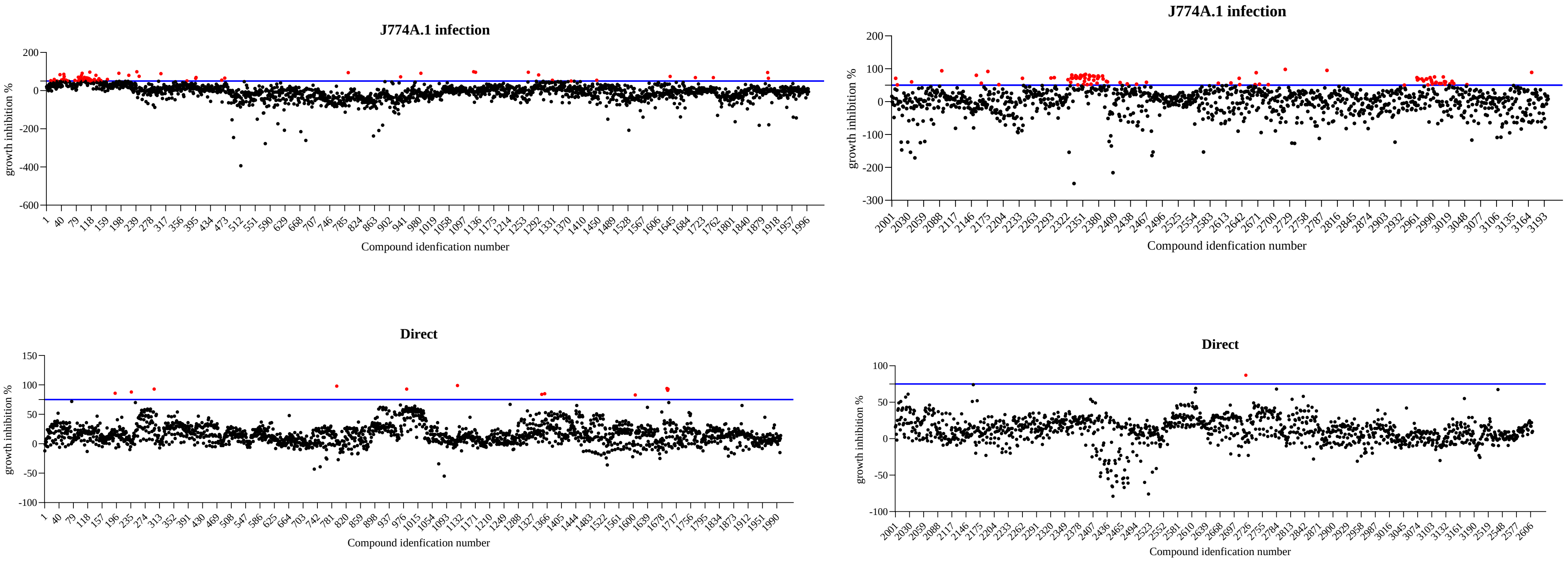

Supplemental Figure 2. Screening results of 3200 clinical compounds. A total of 3200 compounds were applied at a single dose of 5 μM in the direct treatment model and J774A.1 infection model (n=1). Candidates were selected based on an inhibition rate cutoff value of 75% in the direct treatment model and a 50% cutoff value in the J774A.1 infection model. The blue lines represent the cutoff values, and the compounds whose inhibition rates were above the cutoff are shown as red dots.

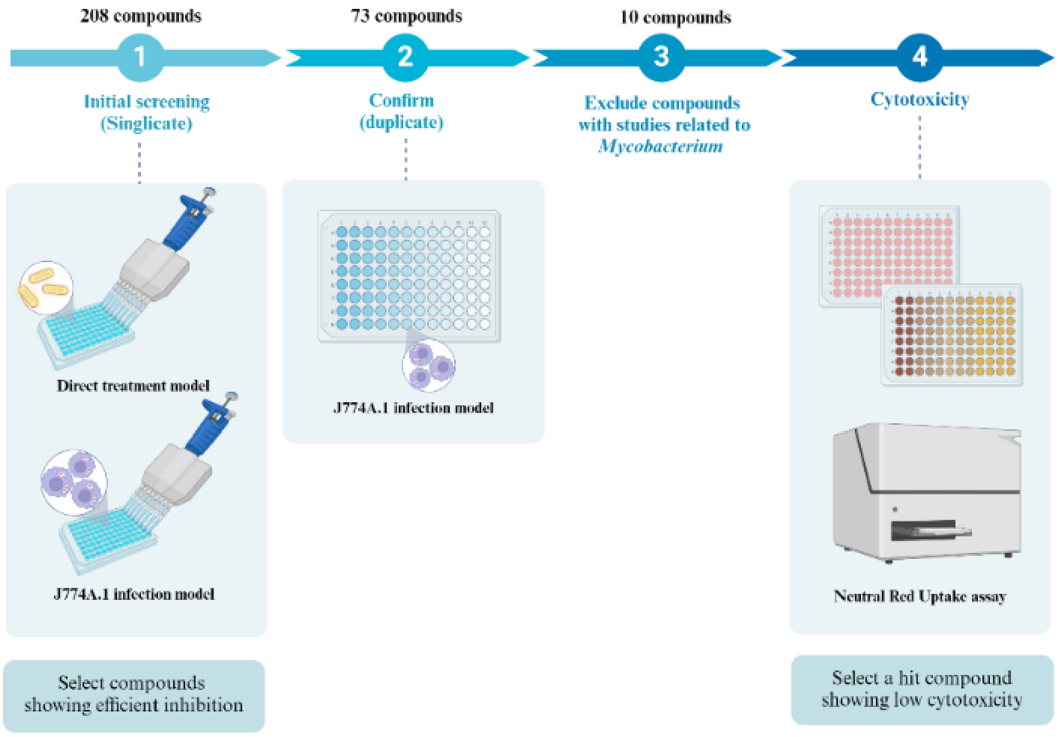

Supplemental Figure 3. Screening procedure of 3200 clinical compounds. Candidates that inhibited more than 75% of growth in the direct treatment model or inhibited more than 50% of growth in the J774A.1 infection model compared with the nontreated control were selected. From this procedure, 208 compounds were identified to inhibit the growth of *M. abscessus*. The antimycobacterial activity of the 208 compounds was subsequently confirmed for validation in duplicate, and 73 compounds were verified to possess antimycobacterial activity. Among these 73 compounds, 10 were selected, excluding 63 compounds that had already been reported as antimycobacterial compounds. Finally, a neutral red uptake (NRU) assay was performed to exclude cytotoxic compounds at effective concentrations, and 8 compounds that exhibited significant cytotoxicity to J774A.1 cells were excluded from the candidates.

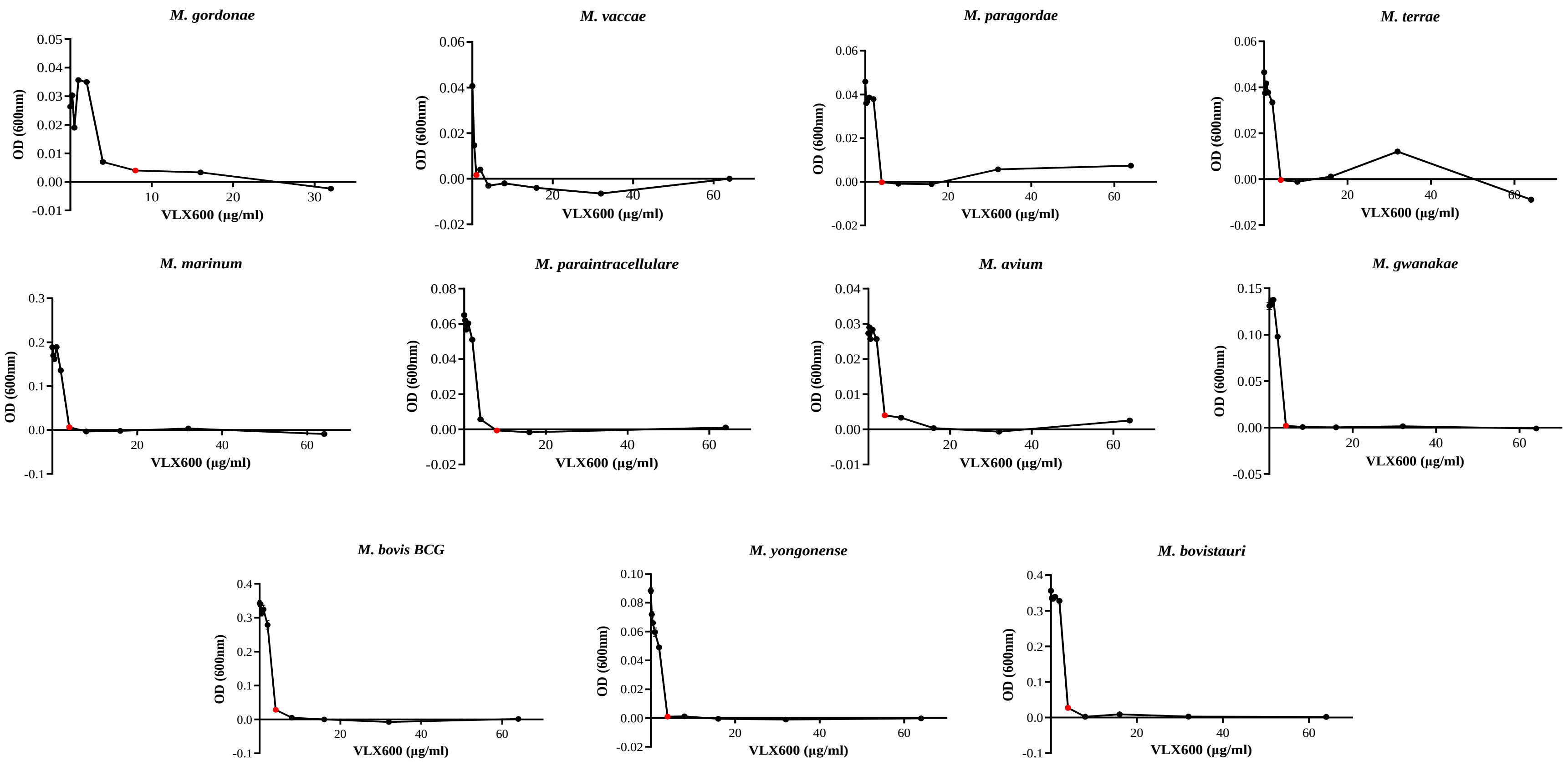

Supplemental Figure 4. 11 species of mycobacteria, including *M. avium*, *M. intracellulare* subsp. *yongonense*, *M. paraintracellulare*, *M. bovis* BCG, *M. gordonae*, *M. paragordonae*, *M. marinum*, *M. vaccae*, *M. terrae*, *M. chleonae* subsp. *bovistauri*, and *M. chelonae* subsp. *gwanakae* were utilized for the MIC test.

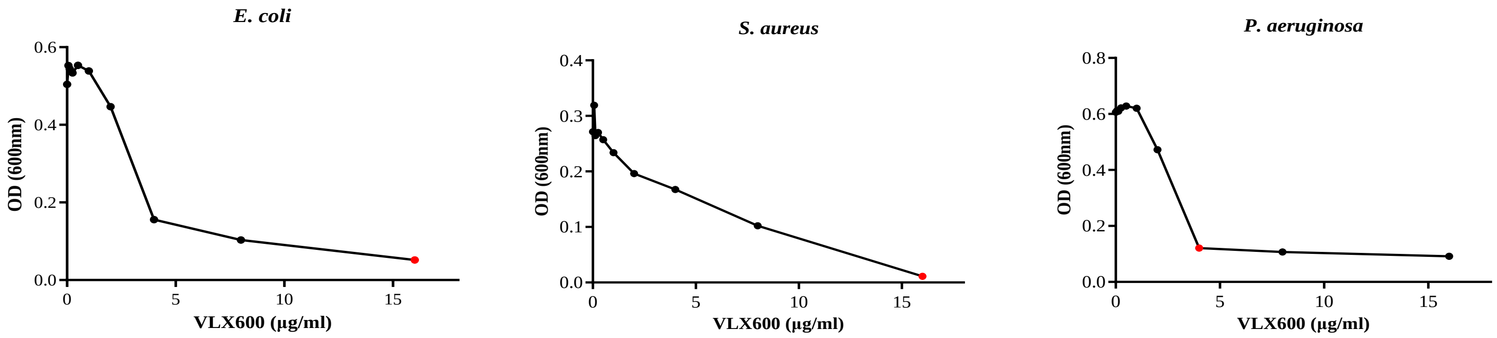

Supplementary Figure 5. Three species of bacteria, including *Escherichia coli*, *Pseudomonas aeruginosa*, and *Staphylococcus aureus* were utilized for the MIC test.

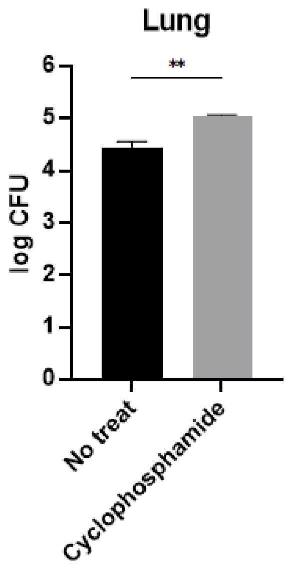

Supplemental Figure 6. The whole lung was excised from each mouse (n=3) and homogenized in PBS. The lung homogenates were serially diluted using appropriate dilution factors and plated on 7H10 agar medium. The plate were then incubated at 37 ℃ until colonies appeared, and the colonies was counted. The statistical significance was determined by two-tailed t test, and the results are denoted as follows: * $p < 0.05$, ** $p <0.01$, and *** $p <0.001$. The error bars represents the standard error of the means.
